# Site-based data curation based on hot spring geobiology

**DOI:** 10.1371/journal.pone.0172090

**Published:** 2017-03-02

**Authors:** Carole L. Palmer, Andrea K. Thomer, Karen S. Baker, Karen M. Wickett, Christie L. Hendrix, Ann Rodman, Stacey Sigler, Bruce W. Fouke

**Affiliations:** 1 The Information School, University of Washington, Mary Gates Hall, Suite. 370 Seattle, Washington United States of America; 2 School of Information Sciences, University of Illinois Urbana-Champaign, Champaign, Illinois United States of America; 3 School of Information, University of Texas at Austin, 1616 Guadalupe Suite #5.202, Austin, Texas, United States of America; 4 Yellowstone Center for Resources, Yellowstone National Park, Yellowstone National Park, Wyoming United States of America; 5 Department of Geology, University of Illinois Urbana-Champaign, Urbana, Illinois United States of America; 6 Department of Microbiology, University of Illinois Urbana-Champaign, 601 S. Goodwin Avenue, Urbana, Illinois United States of America; 7 Carl R. Woese Institute for Genomic Biology, University of Illinois Urbana-Champaign, 1206 W. Gregory Drive, Urbana, Illinois United States of America; 8 Roy J. Carver Biotechnology Center, University of Illinois Urbana-Champaign, 2613 Institute for Genomic Biology, 1206 W. Gregory Drive, Urbana, Illinois United States of America; 9 Thermal Biology Institute, Montana State University, Leon Johnson Hall, Bozeman, Montana, United States of America; Universidade de Vigo, SPAIN

## Abstract

Site-Based Data Curation (SBDC) is an approach to managing research data that prioritizes sharing and reuse of data collected at scientifically significant sites. The SBDC framework is based on geobiology research at natural hot spring sites in Yellowstone National Park as an exemplar case of high value field data in contemporary, cross-disciplinary earth systems science. Through stakeholder analysis and investigation of data artifacts, we determined that meaningful and valid reuse of digital hot spring data requires systematic documentation of sampling processes and particular contextual information about the site of data collection. We propose a Minimum Information Framework for recording the necessary metadata on sampling locations, with anchor measurements and description of the hot spring vent distinct from the outflow system, and multi-scale field photography to capture vital information about hot spring structures. The SBDC framework can serve as a global model for the collection and description of hot spring systems field data that can be readily adapted for application to the curation of data from other kinds scientifically significant sites.

## Introduction

The abundance of publicly accessible scientific data will continue to grow dramatically as the open data movement gains momentum. The potential for open scientific data to spawn new discoveries and innovation has been promoted by dozens of federal reports and active scholarly discourse (e.g., [1–4]), and at present, one international registry lists hundreds of data repositories for the geosciences alone (see www.re3data.org). However, while more and more data has become available, there are still few criteria for guiding the production and management of open datasets to assure their value and fitness for reuse beyond their original application.

The aim of the Site-Based Data Curation project (SBDC) is to develop an approach for retaining the value of digital data collected from scientifically significant sites (hereafter referred to as *site-based data*) for reuse across disciplines. The investigation grew out of our previous work analyzing data curation requirements for more than a dozen scientific subfields [5–6], where site factors emerged as key aspects of data value for researchers in the earth sciences. Sites of data collection are central to many field sciences, as seen for example in the "site-based network approach” applied by the Long-Term Ecological Research (LTER) Network to community-oriented data management at designated biomes, beginning in the 1980s [7]. The SBDC project, funded by the Institute for Museum and Library Services, was motivated by the risks to valid reuse of site-based data through the loss of vital contextual information, as researchers increasingly share their data through institutional and domain repositories, as well as the potential for data services to build rich aggregations of data collected from important research sites.

Using hot springs geobiology research at Yellowstone National Park (YNP) as an exemplar case, we formalized site-based data curation through development of a Minimum Information Framework (MIF) for documenting the critical contextual elements needed for data reuse. Grounded in stakeholder analysis and extensive examination of data artifacts collected by geobiologists, the MIF is designed to function as a guideline for researchers collecting site-based data and as a foundation for further development of curation processes for optimizing the collection, description, and reuse of field data from other kinds of scientifically significant sites.

Data curation is defined as the active management and enhancement of data for current and future use for science, scholarship, and education [8]. This definition assumes the need for both upstream and downstream management of data—from the point of data collection to the archiving and potential enrichment in repositories. Upstream responsibilities generally fall to researchers or research team members responsible for data quality, documentation, and deposit, whereas downstream responsibilities typically fall to information professionals, and range from basic archiving and access work to supporting enhanced interoperability, tools, and services for retrieval, visualization, and analysis. As demonstrated in a recent National Academy of Sciences study [9], demand is growing for data curation and related areas of professional data work in data centers, repositories, libraries, and research institutes. Additionally, organizations and enterprises across the public and private sector are recognizing the importance of investing in the collection, integration, and repurposing data assets [1, 10].

The SBDC project team encompasses the different areas of expertise needed to consider upstream and downstream dimensions of site-based data curation and to address the problem from technical, scientific, and local policy perspectives: 1) data collectors—scientists in geology, microbiology and genomics; 2) site managers—resource managers who oversee data collection at YNP; 3) information scientists specializing in data curation principles and processes; and 4) information professionals with expertise in development of data archiving technologies and data service. The team represents the type of collaboration and division of labor needed to build data systems that retain and add value to data for long-term use and strongly support integrative scientific inquiry.

In the subsections that follow, we first present important background and context, highlighting the integrative geobiology research at Mammoth Hot Springs at YNP that guided the SBDC framework, the primary motivations for the scientific communities, and the related current curation research and development. The second part of the paper describes the participatory methods driving the SBDC project, the empirically derived Minimum Information Framework for geobiology, and discussion of implications and related project themes.

## Background

### Geobiology and Yellowstone

As a mecca for data collection for a range of sciences, YNP serves as an international centerpiece for site-based field research. YNP is a well-protected, well-studied, accessible environment, and the geothermal features in the park are rich with microbial life. The extreme environments found in hot spring vents are used as natural laboratories for investigating research questions that range from the origin and emergence of life on Earth to the search for life on other planets. In particular, Mammoth Hot Springs (MHS) at the northwestern margin of YNP has been the base for decades of integrated geobiological investigations, making MHS a highly suitable location for development of the SBDC framework aimed at supporting systems research that integrates data about geological, biological, physical, and chemical processes.

There are more than 10,000 diverse thermal sites within YNP, allowing geobiology researchers many options for careful study of complex hot spring systems that have not been impacted by human activity and thus remain in their natural state [11]. Furthermore, the sheer number and extreme variety of these thermal environments in YNP allow comparative studies across diverse environmental conditions. This makes it possible to verify system-scale hypotheses regarding the origin and evolution of early life. Over time, potentially significant and important anomalies can be identified from long-term patterns and trends, and then targeted for ongoing and future research.

The National Park Service (NPS) plays a significant role by ensuring that field analyses are completed under stable, consistently accessible, safe, and federally permitted wilderness-sampling conditions. While there are comparable thermal vents found along deep sea spreading centers, their sampling requires complex and expensive logistics (i.e. use of deep water submersibles). With water depths in the kilometers, it is significantly more difficult to discretely sample at a high spatial resolution in the sea than on the land surface. Further, most terrestrial hot springs around the world have not been protected like those at YNP, and therefore research sites that are not heavily damaged and impacted by human use are extremely rare. The relative ease of accessibility of thermal features in their original natural state makes YNP hot springs unique and significant research sites for the geobiological sciences, and YNP an excellent exemplar of a scientifically significant site.

Moreover, as emphasized by our YNP team members, the park is known for forging new benchmarks for professional practice in national parks more generally. For instance, YNP has been an early leader in methods of wildlife and fire management, developing strategies that have been adopted by many other parks (e.g., [12–13]). At this time, YNP is well positioned to lead NPS in areas of data management and curation. The NPS already has processes in place for careful cataloging and tracking of physical specimens and artifacts collected at all national parks [14–15], but there are not yet equivalent protocols for recording, collecting, or preserving digital data. Resource managers at YNP have also taken steps toward better tracking of research in the park to improve monitoring of resources, by building on existing NPS reporting requirements to collect additional information from visiting researchers about their activities in YNP [16]. This emphasis on YNP-specific reporting requirements sets an important precedent for NPS-wide data reporting, and it sets an expectation for documenting and sharing specific information about research activities, consistent with SBDC intentions. It is important to note that internally produced documents and datasets are managed via a suite of NPS-wide systems accessible through the Integrated Resource Management Applications (IRMA) portal [17]. Similar to many institutional repository systems, in its current state IRMA is not designed for robust management of research data. However, it is a serious investment in information infrastructure that over time could evolve to support deposit and retrieval of data content by external researchers.

### Motivations driving SBDC project

From the initial planning stages, the SBDC project was guided by both the scientific aims of academic geobiology researchers external to YNP, and the resource management needs of NPS personnel at YNP. The SBDC core team includes a prominent geobiologist and a YNP senior resource manager, with additional representatives from both groups participating in the stakeholder analysis phase of the investigation. The original design of the project was informed by researcher aims and concerns and further elaborated through systematic engagement throughout the course of the study. Collaborative engagement is a basic tenet of our participatory approach and essential to achieving results that are useful for all stakeholders. The team also built on the experience of existing collaborations between geobiology researchers and NPS personnel, notably the NSF-funded Yellowstone National Park Research Coordination Network (RCN) in which co-PI Fouke was a participant. The scientists participating in the YNP RCN and the SBDC project have demonstrated commitment to data sharing as a way to improve data access and reuse for their common scientific interests in the hot springs environmental context of heat-loving (thermophilic) microbial life.

A key concern in geobiology is the need to understand the environmental context in which early microbial life originated and evolved, but there are few established methods for documenting environmental microbial context. This is largely due to differences in the methodological and disciplinary practices within the core branches of science that contribute to geobiology (biology, physics and chemistry). For example, biology commonly studies cellular to whole organisms contextualized in the environment in which they live. Microbiology, on the other hand, has traditionally pursued research at smaller scales, often analyzing cell physiology, biochemistry, and molecular composition without significant environmental contextualization. This has led to surprisingly limited exchange and cross-referencing between these fields.

The recently developed field of Geobiology [18–19] seeks to better understand complex natural systems by integrating geological, biological, physical and chemical processes with the environmental context in which they occur. This requires the cross-disciplinary integration of: 1) reductionist and holistic approaches; 2) field sampling and laboratory experimentation; and 3) synthesis and prediction across a spectrum of scales of space and time. Ongoing technological advancements in nanometer-scale imaging resolution and high throughput molecular analyses has now made it possible to simultaneously measure, correlate and mechanistically link geobiological processes that range from the single cell to the entire global ecosystem.

In the case of MHS, extensive geobiology research has been completed on how microorganisms respond to, or sometimes rise to control, the coupled effects of environmental change and the deposition of mineral deposits called travertine [20]. For example, MHS thermophiles catalyze the rate at which travertine is precipitated via bacterial cell wall biochemical composition [21]. The hot springs are composed of steep environmental gradients (large physical and chemical changes over short distances) in flow rate, temperature, nutrient delivery, and aqueous chemistry. The thermophiles respond to these changes by changing their bacterial cell wall protein chemistry, which in turn directly controls the rate, shape, and distribution of travertine deposition. As a result, this travertine deposition itself locally changes hot spring outflow environmental conditions, to which cells further respond by changing travertine deposition. This ongoing feedback process creates the terraced travertine morphologies that are the hallmark of MHS, as well as a variety of other changes in the rock deposits. This underlying universal mechanism of microbial catalysis of mineral growth has direct application to understanding biomineralization in the human body (i.e. kidney stone formation), oil and gas exploration (i.e. rock petrophysical properties) and space exploration (i.e. fossilization of ancient mineral biomarkers) [20].

### Related work in data curation and metadata standards

Best practices in scientific data curation have evolved alongside the growth of technical innovations, seen perhaps most vividly in bioinformatics and sequencing technology. The Protein Data Bank and GenBank have become canonical models of success in the curation of large aggregations of shared scientific data [22–23]. For the sciences that have been developing protein and gene data systems, curation work has largely focused on annotation and assembly of metagenomes and genomes, with only minimal, if any, requirements for contextualization of the data collection environment. However, as discussed below, for utilization of GenBank data by geobiology researchers, the curation process would need to also produce metadata documenting system-level information on the site where genetic data were collected.

Metadata is a central concern in data curation, since structured descriptive information is essential for discovery of content in information systems and for users to understand the potential for data to be reused. At the most basic level, metadata for a dataset should capture “who, what, where, when, and how” data were collected, but further information about measurements and definitions of variables and units is necessary for data to be usable by anyone beyond the original collector [24]. Studies in the earth sciences have emphasized the importance of documenting methods and protocols, especially since environmental conditions and disturbances in the field can result in unexpected changes in procedures [7, 25–26].

Metadata standards in the earth sciences have varying and often limited coverage of data collection methods [27]. Many include an element for “sampling” and optional description of summary elements that can accommodate some unstructured aspects of methods description. The Ecological Metadata Language (EML) takes a relatively comprehensive approach distinguishing between protocols and methods, and with structured sub-elements for methods procedures that includes temporal and/or spatial coverage and instruments used in specific processes. Studies of data practices, however, have shown that many researchers are not aware of or do not implement the metadata standards in their discipline [28], in some cases because of their complexity or a need for technical support [29]. Nonetheless, standards for specific areas of research and data types continue to proliferate. For example, Biosharing (https://www.biosharing.org/standards/), a resource focused on life sciences data standards, databases, and policies, lists more than 600 standards. Notably, Biosharing includes over 80 reporting guidelines, which have been largely drawn from an initiative working to harmonize minimum information standards, called Minimum Information about a Biomedical or Biological Investigation (MIBBI) [30].

It was not the goal of the SBDC project to create a metadata standard for geobiologists, but rather to create a framework of principles and processes that helps to articulate and support upstream and downstream processes as a general model for site-based data curation. Early work with our participants, however, suggested that a minimum information framework (MIF) would be a productive step for our stakeholder communities and consistent with trends in other sciences. A MIF can offer basic structure for documenting data collection processes, as well as a way to extend current schemas that lack context description.

## Methods: Stakeholder analysis and engagement

The curation framework was developed primarily through iterative phases of stakeholder analysis and participatory engagement, drawing on principles from natural resource management [31–32] and methods adapted from our previous data practices research [33–34]. Our stakeholder analysis approach is rooted in the Delphi technique that enlists a panel of experts to solve problems through a process of consensus development [35]. It was also helpful in building a sense of community among the participants with varying interests and needs in accessing YNP data. Further, as reviewed in [36] a participatory approach to standards development is crucial for long-term adoption, particularly in scientific contexts. We have also closely monitored community developments of the National Geothermal Data System [37] and the Yellowstone Research Coordination Network [38], which contain stores of data relevant to emergent SBDC principles and practices, and consulted with developers of the National Environmental Methods Index.

We began the process with a *stakeholder workshop* held at YNP in Spring of 2013. Through this workshop, we gained further understanding of our stakeholders' practices in the field, expectations for data quality and reusability, opinions regarding data sharing, and initial criteria for a minimum information framework. The MIF was then further elaborated and refined through *participatory engagement* and through several kinds of *artifact analysis* conducted with a large sample of YNP geobiology data.

### Stakeholder workshop

A two-day workshop held at YNP served as the foundation for engagement with two key groups: academic scientists who collect data at YNP and, resource management personnel from the park. Participants included nine scientists—geologists, geochemists, and microbiologists—who have research programs dependent on data they collect at YNP, and seven professionals from YNP, including managers of research permitting and reporting, and information professionals from the YNP research library and archive. The workshop was designed to interrogate data value and reuse factors through a set of roundtables, exercises, and focus groups with the researchers and YNP personnel. The set of activities generated a shared understanding of broad curation goals, and revealed that researchers and resource managers had different priorities and functional requirements for YNP data. Generally, researchers wanted curation designed to optimize data for longitudinal studies across sites and exploration of the dynamic systems within YNP. Resource managers at YNP were particularly interested in using data sources to track site evolution over time, and in using metadata to inform strategic science initiatives in the park. All participants agreed that well-curated and aggregated YNP data will be critical to making important scientific advances in understanding microbial metabolic function, their linked relationship to the environment, and the evolution of these thermophilic communities in space and time.

Participants also considered the strengths and limitations of the standards that currently guide their own data collection, including: sampling protocols, approaches to field notebook data entry, trip logs, and other descriptive techniques used in data collection, processing, and analysis. An important area of consensus was the vital role of photographs as metadata for documenting site conditions, environmental context, and geologic setting associated with sample collection. Field photographs were also seen as an important means for tracking seasonal and other changes at a site over time. As one researcher noted, "The first thing I want to know about a dataset is, *'What did the spring look like*?*' "*

Transcripts of the focus groups and extensive notes from the other sessions were inductively coded to identify key themes and requirements, recorded along with a full account of the workshop activities in the final report [39–40]. The University of Illinois at Urbana-Champaign Institutional Review Board (IRB) approved this research. Written consent was obtained from our participants prior to beginning research. The IRB approved this consent procedure.

### Participatory engagement

Participatory engagement and artifact analysis after the workshop focused on MIF development and refinement. The preliminary list of data elements to be included in the MIF was drafted by two of the geobiologists immediately following the workshop. These terms were first revised based on the analysis of the workshop outcomes and then through consultation and interactions with selected key stakeholders (three geobiologists and two representatives from YNP) over the next year. Interactions included interviews, email exchanges, conference calls, and face-to-face meetings.

Direct analysis of data artifacts was used to assess the efficacy and coverage of the MIF. First, we conducted an extensive inventory of co-PI Fouke's research hard drive, in which we modeled his typical research workflows and data outputs based on two years' worth of his data. In addition to a data inventory, the artifact analysis produced a process inventory, activity diagram, and provenance graph, to be reported on in depth in subsequent publications. The focused case study of a significant personal collection of YNP data gave us valuable perspective on a typical research workflow and data organization processes, and it verified the saliency of the MIF elements for the research process and data products. This work will be described in detail in another publication.

The MIF was also compared with an existing data publication standard identified from a list of relevant initiatives and platforms developed through stakeholder analysis and a survey of the literature. The EarthChem "Vent Fluids" template [41] was found to be the most applicable to the kinds of chemical data recorded in geobiology research [42]. Finally, we conducted a trial run in the field using a custom data entry template rooted in the MIF for co-PI Fouke's undergraduate "Introduction to Biocomplexity" class trip to YNP to learn about fieldwork. Students were observed entering data into the template, and their experiences and feedback informed our final revisions of the MIF.

The MIF and the results of these latter two applications of the MIF are described in the following section. It has been conceived of as a guideline or structured set of expectations that can serve as a basis for information models and support further development and practical application by the geobiology community. As noted above, the participatory methods applied are particularly appropriate for this work, since standards are most effective when developed and enforced by the community they are meant to support [36].

## Results

The results from the stakeholder analysis guided the design of the SBDC MIF. A range of themes emerged from the various activities, representing the different perspectives of the scientific disciplines and YNP management. In terms of the primary SBDC aim of supporting the reuse of data across research communities, two priorities were clear. There was consensus among the participants that the potential for valid use of data produced by someone else depends on: 1) documentation of sampling procedures, and 2) contextual information about the site of data collection. Through the iterative MIF development we engaged with participants and examined data collections, working toward agreement on how to represent these two categories of information as efficiently as possible. Reframing the aims around capturing “minimum information” rather than production of metadata was consistent with data standards trends in biology. It also allowed us to avoid a common (and in this case, unproductive) distinction between “metadata” and “data,” since we determined that the information needed to represent sampling and site context is a mixture of qualitative descriptive elements and measured data points.

Grounded in a “systems science” understanding of YNP hot springs, one of the most novel contributions of the MIF schema is the assertion that both quantitative and qualitative information about both the hot spring vent and the outflow drainage system, and the relationship between them, are required. Each of these components is fundamental to understanding the dynamic structure and physical, chemical and biological composition of any given hot spring. The geobiologists noted that studies tend to provide various degrees of information on one or the other but rarely for both the vent and the outflow. For the purposes of sharing hot spring data, they determined that description of the vent as well as the drainage system are vital for contextualizing samples and measurements within the broader system, to ensure accurate and valid integration and reuse of the sample data.

### Minimum information framework

**Fig 1:**
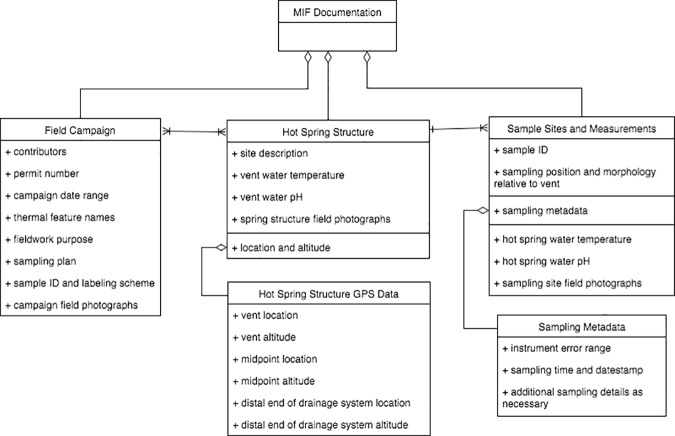
UML Class Diagram showing the three classes of the Minimum Information Framework (MIF): Field Campaign, Hot Spring Structure and Observations from Sample Sites.

The MIF is composed of three classes of information (Fig 1). The Field Campaign class provides basic information about the people involved in a project, the project's mission, and the springs being studied. This includes a description of the *fieldwork purpose* articulating overall goals and motivating hypotheses; any related *NPS permit numbers*; *names* of people who collected the data in the field; *date range* of the project; *sampling plan* for data collection; explanation of the *sample ID and labeling schema* (e.g., explanation of any codes used to label samples); and *names of hot springs* being sampled (specific geolocations of the springs are required as well but documented under a different class). Large-scale photography of the entire hot spring system should be included here.

The Hot Spring Structure class includes information that describes and characterizes each hot spring within the study. Special focus is paid to the vent of the hot spring, which serves as the triangulation point for all of the outflow drainage system. The vent *temperature* and *vent pH* of the water at the vent should be recorded; this acts as a heuristic characterization of the hot spring's microbial ecology [20]. Other information elements include a *site description* in free text (accompanied by sketches if necessary); and the *location and altitude* of the *vent*, the *mid-point* of the drainage system, and the *end-point* of the drainage system. This array of points would allow the geometry and flow directions of the entire system to be identified and reconstructed. Detailed *photographs of the entire spring system* are included to clearly illustrate the spatial hydrologic continuity between the vent and the outflow drainage channel.

The Sample Sites and Measurements class includes information about each site of sample collection. *Sample ID* is the label assigned to the sample or measurement; this is critical for capturing the provenance of future analyses. *Sampling position* should describe the position of each sampling site in terms of the distance and bearing to the vent, and the *morphology* of the lithographic facies the sampling site is located in (e.g., the pond, apron or channel of the hot spring drainage system; see [20]). We note that a description of distance and bearing is necessary in lieu of a simple GPS location because the distance between sampling sites (often a few centimeters to meters) in these springs is often too small to be recorded accurately by GPS. Any relevant instrument-specific *sampling metadata* should also be recorded (e.g., error ranges for thermometers, date and timestamps of measurements). The sample site's water *temperature* and *pH* should be collected (preferably in triplicate) along with each sample. Detailed photographs at a range of scales should be included, to clearly illustrate the spatial relationships and hydrologic continuity between each sample collection site and its position in the vent and the outflow drainage channel and collection sites therein.

**Fig 2:**
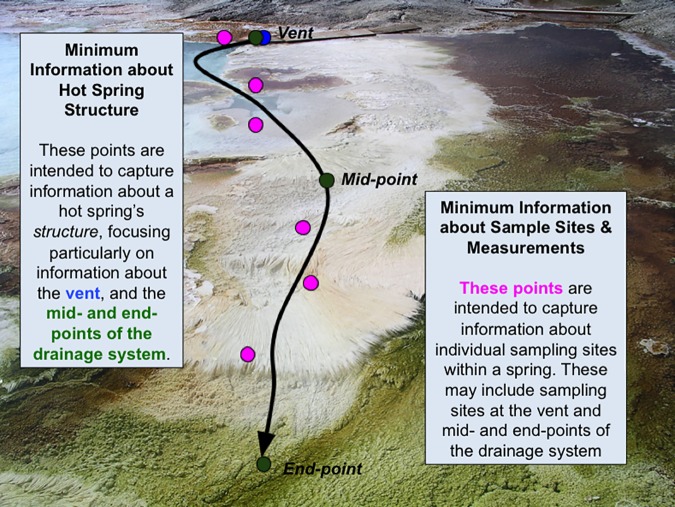
Illustration of geospatial relationship between information collected in the Hot Spring Structure class and the Observations from Sample Sites class. Photograph taken by Bruce Fouke and previously published in [20].

In Fig 1, it is important to note the many-to-many relationship between field campaign and hot spring structure (many field campaigns can study many hot springs and many hot springs can be studied in many field campaigns) and the one-to-many relationship between hot spring structure and sample sites. The Hot Spring Structure GPS data is split out as a subclass of Hot Spring Structure location and altitude data. Fig 2 illustrates the relationship between these latter two classes of information. Sampling Metadata is split out as a subclass of the Sample Sites and Measurements class because of it's great variability; we anticipate that researchers seeking to apply the MIF to their work would need to customize this class to their study and instrumentation. Researchers may wish to include information on sampling technique and experimental design, measurement units and uncertainty, and instrument detection limits in this subclass.

### Encoding guidelines

The MIF is intended to guide general documentation practices for sample and data collection for geobiology. As such, the UML class diagram shown in Fig 1 should act as a starting point for developing documentation and information management practices that are appropriate for a given field campaign. The MIF classes and attributes should be refined based on the sampling plans and instrumentation developed for the field campaign. Additionally, research communities will need to strive for systematic encoding of the MIF elements, with initial recommendations as follows. For the Field Campaign elements, *Contributors* should include the full name(s) of PI/Investigators and all members of the field party. *Thermal Feature Names* lists the names of locations where sampling was conducted. For sites at YNP, use of the "official" location names and ID numbers found in the NPS Thermal Inventory is recommended if known, (though we note that corrections may be needed depending on changes in thermal feature activity over time). *GPS locations* should be recorded in UTM if possible. *Altitude* measurements should be represented in meters, and derived from topographical maps or separate GPS systems. *Photographs* should be taken at a range of scales for each class of information (mm-cm-mm length), preferably with embedded geolocation and timestamp information. *Site descriptions* should detail the overall site and condition of the vent and sampling sites, as well as information describing the primary flow path in a range of methods (e.g., sketches, free text description, estimated size of spring, etc.)

Controlled vocabularies need to be applied when possible. As noted there are official names for YNP geological features (e.g., Angel Terrace), but sub-feature terms, such as the names of the facies (e.g., pond, apron, channel, etc.) along the spring drainage system, should also be consistently applied. This may involve development of a local controlled vocabulary. Other controlled vocabularies may need to be adapted or extended. For example, terms from the Biodiversity Collections Ontology [43] may be appropriate for description of sampling plans and locations, and terms from the growing the National Environmental Methods Index [44] may be useful in describing sampling methods. For some sampling methods, a simple description of what kind of instrumentation was used may suffice, such as “by paper” or “by instrument” for pH measurement.

### Evaluation

The applicability of the MIF was refined and tested in two ways, through: 1) comparison to existing standards, focusing on EarthChem Vent Fluids templates, and a 2) trial run in the field with co-PI Fouke's undergraduate "Introduction to Biocomplexity" class trip to YNP.

To assess the MIF's compatibility with existing data publishing infrastructures, we examined the coverage and fit in relation to the following controlled vocabularies and data standards: US Geological Survey, Geographic Names Information System; National Environmental Methods Index; Marine Geoscience Data System; Federal Geographic Data Committee geo-metadata standard; ISO19115; and the Integrated Earth Data Application, EarthChem data templates. Of these community-developed standards, we found that the EarthChem “Vent Fluids” template [41] was best for capturing the nuances within the extensive samples of geobiology data. It too features a hierarchical structure that captures information about a field campaign, methods metadata, and site and sample context (including a "lithology of substrate field" which could be used to express the morphology of hot spring facies at each sample site). As would be expected, many aspects of the MIF specific to hot springs did not "fit" into the existing template, such the sampling position and morphology relative to the vent. If the MIF were to be adopted by a broader community, however, the existing EarthChem "Vent Fluids" template would be an excellent foundation for adapting a hot spring-specific data template. This work is described further in [42].

To assess the application of the MIF in practice, the SBDC team tested a prototype of the framework with a group of students in co-PI Fouke's Introduction to Biocomplexity course, in which students learn to collect data in the field. We created a spreadsheet template formatted for the students' data collection based in the MIF for use on a field trip to YNP where they conducted small experiments and data collection in the hot springs. After training in data curation best practices and use of the template provided by an SBDC team member, the students used the templates for their data collection and field work. Based on direct observation of the students' work in the field and review of their completed data collection sheets, the MIF functioned well in supporting structured description of data at the point of collection in the field. Some students reported that they enjoyed the template description process, and none found it to impede their project work. However, some had difficulty with precise description of the vent location and the bearing of each of their sample sites relative to the vent, due to the complexity and access limitations of the data collection location. Students were working in the main area of Mammoth Hot Springs, which includes upwards of 20 individual vents and several overlapping springs. They were consequently restricted to the "boardwalk" paths and unable to reach certain segments of the springs. We discuss the implications of this trial run for future iterations and uses of the MIF below.

## Discussion

The MIF is rooted in geobiologists’ “state-of-the-art” understanding of how best to parameterize YNP hot springs as a holistic, complex natural system. It offers data curation guidance well beyond the *post hoc* description of a data product typical of many descriptive metadata approaches, and includes context and relative measurements essential to retaining the value and usability of the data for earth systems geobiology. Information describing the vent, for example, serves as a critical anchor-point for measurements at a certain point in time and accommodates the need to account for change in natural systems as part of data stewardship responsibilities. The MIF prioritizes data curation best practices of documenting provenance as well as the relationships among related data objects and their context. The unique contributions of the MIF include the three classes of elements, reflecting fundamental levels of representation of the process of collecting data in the field, and the utilization of photographic information as a formal part of the descriptive scheme.

Implementation of the MIF has a number of challenges. The time and labor involved in data documentation and description is widely acknowledged as a general limitation in the generation of metadata. A successful implementation strategy would need to build on existing reporting procedures and practices to extend the “small structures” that are common in the research process, such as existing data management routines and reporting requirements for research conducted in national parks. An important next step would be the development of a metadata tool with menus of standard protocols for encoding MIF elements and forms that prepopulate fields with information on methods regularly applied by a data collector. Additionally, data processing tools need to support dataset normalization, packaging, and description of complex data objects. At the same time, these techniques should not inhibit individualized methods for sampling, experimentation, analysis and computation.

As revealed through our trial run with students in the Biocomplexity class, even in localities as accessible as YNP, there are challenges in gaining sufficient access to the site for recording some elements. In large hot springs, researchers may not be able to precisely locate key structural components, such as the vent or the mid- and end-point of the drainage channel, and they may not be able to precisely measure the position of sampling sites relative to the vent. In such cases, estimates of distances would be a reasonable alternative for supporting later reconstructions of the hot spring structure. Additionally, since standard geolocation points can be less than optimal, supplemental photography (including recent satellite imagery) and field sketches should be encouraged. As noted earlier, we thus recommend that researchers create both qualitative and quantitative representations of the hot springs and their sampling sites, thereby creating field data that can be reused and integrated for systems science.

### Aggregation of site-based data

The geobiologists engaged in this study were highly aware that they need to be able to gather site-based data for to conduct sophisticated system level analyses. At present they have limited ability to examine the complex big picture of natural systems, determine normal conditions over time, conduct broad comparative studies, or reveal new connections and relations among isolated studies. The research community at present seems to have a culture of collective autonomy; they are highly aware of each other’s work in YNP and the particular kinds of data collected from the same sites over years or decades, but they do not work together collaboratively or coordinate their collective data resources.

While the MIF is an important step in supporting reuse of site-based data, metadata alone does not directly address researchers’ need for access to site-based YNP data in aggregate. Tools and infrastructures are needed to support site-based data curation. Integration of data from particular sites or comparison across sites over time would be supported by consistent community application of the MIF as well as systems or repositories that aggregate the standardized data and allow search and retrieval within and across sites. Map-based interfaces are becoming more common as an access layer in geoscience data systems, but most disciplinary and institutional data repositories do not support discovery of data based on scientifically significant sites.

Resource managers in the park also expressed interest in a system that would support systematic and comprehensive understanding of the data generated at specific locations. Site-based monitoring of data collection could be used to mitigate risk of redundant or excessive work in delicate ecological areas. As part of the permitting process, resource managers could also assist scientists in identifying areas with potential for high impact data for their research questions and other existing data from those sites. An SBDC approach to data description and aggregation could support and enhance a range of reporting requirements, such as the annual reporting required of researchers with NPS data collection permits, but also the data management plans required by funding agencies.

Current systems outside the NPS where YNP geobiology data could be deposited and aggregated capture some of the elements of the SBDC framework in different ways and in varying degrees. Data made available by the YNP Research Coordination Network [38], for instance, may be accompanied by a photo to provide some indication of site conditions at the time of fieldwork; latitude and longitude coordinates, geothermal region, and complex area may also be provided, as well as pH, and temperature. However, data collection procedures are not typically described. The Geothermal Data System [37] applies a number of appropriate standards, including ISO 19115 and a mandatory “lineage” field, “based on sources which are either used or produced in a series of process steps,” but it does not provide structure for detailed specifications on thermal features and conditions. The System for Earth Sample Registration [45], designed for physical samples, has developed its own metadata profile that includes a “collection methods” field in the general description element as well as a site geolocation element and fields such as "Physiographic Features." However, the initiative is still in the early phase of developing an approach for connecting to digital data. The SBDC empirically derived MIF is distinctly different from these and other existing site-based standards. By distinguishing the field campaign, hot spring structure, and sample site as distinct classes and anchoring data around characterizations of the hot spring vent, it would function well as a formal foundation for extending the schemas used in these existing earth science systems.

## Conclusion

The current SBDC framework has been developed for geobiology research in hot springs and is informed by both participant needs and an understanding of how hot springs are structured and function. In the next phase of SBDC, the team intends to investigate and test how readily the MIF can be adapted to a different type of geologic feature and the associated needs of the research community. We expect that certain aspects of sampling and site context may be similar. However, the measurement anchors, application of images, and contextual parameters may be unique. For instance, we are considering a study of coral reef science for our next case study; in this science, seawater depth (bathymetry) and distance from shore would likely replace hydrological flow path from vent through the drainage system. Like the work with the hot springs, any future MIF development for coral reefs or any other scientifically significant site will be guided by work with the scientific community and a scientific understanding of how to retain the value of data and integrate different kinds of data from a given site as a long-term data stewardship responsibility.

## References

[1] National Science Board. Long-lived digital data collections enabling research and education in the 21st century. [Internet] 2005. Available from: https://www.nsf.gov/geo/geo-data-policies/nsb-0540-1.pdf

[2] National Science and Technology Council. Harnessing the Power of Digital Data for Science and Society: Report of the Interagency Working Group on Digital Data to the Committee on Science of the National Science and Technology Council [Internet]. [cited 2016 Nov 24]. Available from: http://www.nitrd.gov/About/Harnessing_Power_Web.pdf.

[3] Committee on Strategy and Budget Task Force on Data Policies; National Science Foundation Task Force on Data Policies. Digital Research Data Sharing and Management [Internet]. National Science Board. 2011. Available from: https://www.nsf.gov/nsb/publications/2011/nsb1124.pdf

[4] Hey T, Tansley S, Tolle KM. The fourth paradigm: data-intensive scientific discovery Redmond, WA: Microsoft research; 2009 10.

[5] Cragin MH, Palmer CL, Carlson JR, Witt M, A PTRS. Data sharing, small science and institutional repositories. Philos Trans R Soc A Math Phys Eng Sci. 2010;368:4023–38. 10.1098/rsta.2010.0165 20679120

[6] Palmer CL, Weber NM, Cragin MH. The analytic potential of scientific data: Understanding re‐use value. Proc Am Soc Inf Sci Technol [Internet]. 2011 [cited 2012 Mar 20].

[7] Karasti H, Baker KS. Digital Data Practices and the Long Term Ecological Research Program Growing Global. Int J Digit Curation [Internet]. 2008;3(2):42–58. Available from: http://ijdc.net/index.php/ijdc/article/view/86

[8] Cragin MH, Heidorn PB, Palmer CL, Smith LC. An Educational Program on Data Curation. Poster presented at American Library Association Conference, Science and Technology Section. 2007 Jun 25. Washington, D.C.

[9] National Research Council. Preparing the Workforce for Digital Curation [Internet]. 2015. 91 p. Available from: http://www.nap.edu/download.php?record_id=18590# 25950079

[10] Curry E, Freitas A, O’Riáin S. The Role of Community-Driven Data Curation for Enterprises In: Wood D, editor. Linking Enterprise Data. Springer; 2010 p. 1–291.

[11] Hydrothermal Features—Yellowstone National Park (U.S. National Park Service) [Internet]. [cited 2016 Nov 24]. Available from: https://www.nps.gov/yell/learn/nature/hydrothermal-features.htm

[12] Wright RG. Wildlife management in the national parks: questions in search of answers. Ecological Applications. 1999 2 1;9(1):30–6.

[13] Turner MG, Romme WH, Tinker DB. Surprises and lessons from the 1988 Yellowstone fires. Frontiers in Ecology and the Environment. 2003 9 1;1(7):351–8.

[14] National Park Service—Museum Management Program [Internet]. [cited 2016 Nov 24]. Available from: https://www.nps.gov/museum/publications/handbook.html

[15] Mandatory Curatorial Responsibilities of Research Permit Holders—Yellowstone National Park (U.S. National Park Service) [Internet]. [cited 2016 Nov 24]. Available from: https://www.nps.gov/yell/learn/management/curatorial.htm

[16] IRMA Portal [Internet]. [cited 2016 Nov 24]. Available from: https://irma.nps.gov/Portal

[17] Yellowstone Permit Conditions—Yellowstone National Park (U.S. National Park Service) [Internet]. [cited 2016 Nov 24]. Available from: https://www.nps.gov/yell/learn/nature/ynpconditions.htm

[18] ASM. Geobiology: Exploring the Interface Between the Biosphere and the Geosphere, American Academy of Microbiology, Washington DC. 2001. 57 pp. 32687284

[19] Agouron. Geobiology: Current Technology and Resource Needs, Agouron Institute, Pasadena. 2010. 43 pp.

[20] Fouke BW. Hot-spring Systems Geobiology: abiotic and biotic influences on travertine formation at Mammoth Hot Springs, Yellowstone National Park, USA. Sedimentology [Internet]. 2011 1 17 [cited 2014 Jul 25];58(1):170–219.

[21] Kandianis M.T., Fouke B.W., Veysey J., Johnson R.W. and Inskeep W. Microbial biomass: a catalyst for CaCO3 precipitation in advection-dominated transport regimes. Geological Society of America Bulletin. 2008:120, 442–450.

[22] Berman H, Henrick K, Nakamura H, Markley JL. The worldwide Protein Data Bank (wwPDB): ensuring a single, uniform archive of PDB data. Nucleic acids research. 2007 1 1;35(suppl 1):D301–3. 17142228 10.1093/nar/gkl971 PMC1669775

[23] Howe D, Costanzo M, Fey P, Gojobori T, Hannick L, Hide W, Hill DP, Kania R, Schaeffer M, St Pierre S, Twigger S. Big data: The future of biocuration. Nature. 2008 9 4;455(7209):47–50. 10.1038/455047a 18769432 PMC2819144

[24] Fegraus EH, Andelman S, Jones MB, Schildhauer M. Maximizing the Value of Ecological Data with Structured Metadata: An Introduction to Ecological Metadata Language (EML) and Principles for Metadata Creation. The Bulletin of the Ecological Society of America [Internet]. 2005 7 1 [cited 2016 Nov 24];86(3):158–68.

[25] Mayernik MS, Wallis JC, Pepe A, Borgman CL. Whose data do you trust? Integrity issues in the preservation of scientific data. Proceedings of the 2008 iConference. 2008. Available from http://hdl.handle.net/2142/15119

[26] Karasti H, Baker KS, Halkola E. Enriching the Notion of Data Curation in E-Science: Data Managing and Information Infrastructuring in the Long Term Ecological Research (LTER) Network. Comput Supported Coop Work [Internet]. 2006 8 1 [cited 2016 Nov 24];15(4):321–58. Available from: http://link.springer.com/article/10.1007/s10606-006-9023-2

[27] Chao TC. Enhancing metadata for research methods in data curation. Proc Am Soc Info Sci Tech [Internet]. 2014 1 1 [cited 2016 Nov 24];51(1):1–4.

[28] Tenopir C, Dalton ED, Allard S, Frame M, Pjesivac I, Birch B, et al Changes in Data Sharing and Data Reuse Practices and Perceptions among Scientists Worldwide. PLOS ONE [Internet]. 2015 8 26 [cited 2016 Nov 24];10(8):e0134826 Available from: http://journals.plos.org/plosone/article?id=10.1371/journal.pone.0134826 10.1371/journal.pone.0134826 26308551 PMC4550246

[29] Mayernik MS, Batcheller AL, Borgman CL. How Institutional Factors Influence the Creation of Scientific Metadata. In: Proceedings of the 2011 iConference [Internet]. New York, NY, USA: ACM; 2011 [cited 2016 Nov 24]. p. 417–425. (iConference ‘11).

[30] Taylor CF, Field D, Sansone S-A, Aerts J, Apweiler R, Ashburner M, et al Promoting coherent minimum reporting guidelines for biological and biomedical investigations: the MIBBI project. Nat Biotechnol [Internet]. Nature Publishing Group; 2008 8;26(8):889–96. 10.1038/nbt.1411 18688244 PMC2771753

[31] Grimble R, Wellard K. Stakeholder methodologies in natural resource management: a review of principles, contexts, experiences and opportunities. Agricultural systems. 1997 10 31;55(2):173–93.

[32] Reed MS, Graves A, Dandy N, Posthumus H, Hubacek K, Morris J, Prell C, Quinn CH, Stringer LC. Who's in and why? A typology of stakeholder analysis methods for natural resource management. Journal of environmental management. 2009 4 30;90(5):1933–49. 10.1016/j.jenvman.2009.01.001 19231064

[33] Cragin MH, Chao TC, Palmer CL. Units of Evidence for Analyzing Subdisciplinary Difference in Data Practice Studies. In: Proceedings of the 11th Annual International ACM/IEEE Joint Conference on Digital Libraries [Internet]. New York, NY, USA: ACM; 2011 [cited 2016 Nov 24]. p. 441–442. (JCDL ‘11).

[34] Chao TC, Cragin MH, Palmer CL. Data Practices and Curation Vocabulary (DPCVocab): An Empirically Derived Framework of Scientific Data Practices and Curatorial Processes. J Assoc Inf Sci Technol. 2015;66(3):616–33.

[35] Sackman H. Delphi Assessment [Internet]. Santa Monica, CA: RAND Corporation; 1974 [cited 2016 Nov 24]. Available from: http://www.rand.org/pubs/reports/R1283.html

[36] Yarmey L, Baker KS. Towards Standardization: A Participatory Framework for Scientific Standard-Making. Int J Digit Curation [Internet]. 2013;8(1):157–72. Available from: http://www.ijdc.net/index.php/ijdc/article/view/252

[37] National Geothermal Data System (NGDS) [Internet]. [cited 2016 Nov 24]. Available from: http://geothermaldata.org/

[38] Yellowstone Research Coordination Network [Internet]. [cited 2016 Nov 24]. Available from: http://www.rcn.montana.edu/

[39] Thomer AK, Palmer CL, Wickett KM, Baker KS, Jett JG, Dilauro T, et al. Data Curation for Geobiology at Yellowstone National Park: Report from Workshop Held April 16–17, 2013 [Internet]. 2014. Available from: http://hdl.handle.net/2142/47070

[40] Palmer CL, Thomer AK, Baker KS, Wickett KM, Varvel V, Choudhury S, et al Building a Framework for Site-Based Data Curation. Proc ASIST 2013 [Internet]. 2013;(50):1–4.

[41] Data Templates | EarthChem [Internet]. [cited 2016 Nov 24]. Available from: http://www.earthchem.org/data/templates

[42] Gordon SC, Thomer AK, Dilauro T, Jett JG, Fouke BW, Palmer CL. Site Based Data Curation: Developing a Data Portal for Geobiologists at Yellowstone National Park. Proc Am Soc Inf Sci Technol. 2014;51:1–4.

[43] Walls RL, Deck J, Guralnick R, Baskauf S, Beaman R, Blum S, et al Semantics in Support of Biodiversity Knowledge Discovery: An Introduction to the Biological Collections Ontology and Related Ontologies. Bajic VB, editor. PLoS One [Internet]. 2014 3;9(3):e89606 Available from: http://dx.plos.org/10.1371/journal.pone.0089606 10.1371/journal.pone.0089606 24595056 PMC3940615

[44] National Environmental Methods Index [Internet]. [cited 2016 Nov 24]. Available from: https://www.nemi.gov/home/

[45] Welcome to SESAR, the System for Earth Sample Registration | System for Earth Sample Registration [Internet]. [cited 2016 Nov 24]. Available from: http://www.geosamples.org/.

